# Differential analysis of microbiomes in mucus and tissues obtained from colorectal cancer patients

**DOI:** 10.1038/s41598-022-21928-4

**Published:** 2022-10-28

**Authors:** Yosuke Tajima, Shujiro Okuda, Tsunekazu Hanai, Junichiro Hiro, Koji Masumori, Yoshikazu Koide, Tadahiro Kamiya, Yeongcheol Cheong, Gaku Inaguma, Yoshifumi Shimada, Toshifumi Wakai, Hayato Takihara, Shingo Akimoto, Hiroshi Matsuoka, Ichiro Uyama, Koichi Suda

**Affiliations:** 1 Department of Gastrointestinal Surgery, Fujita Health University, 1-98 Dengakugakubo, Kutsukake, Toyoake, Aichi 470-1192 Japan; 2 Division of Digestive and General Surgery, Niigata University Graduate School of Medical and Dental Sciences, Niigata, Japan; 3 Medical AI Center, Niigata University School of Medicine, 2-5274 Gakkocho-dori, Chuo-ku, Niigata, 951-8514 Japan

**Keywords:** Bacteria, Colorectal cancer, Microbiology, Gastroenterology

## Abstract

The outer mucus layer of the colorectal epithelium is easily removable and colonized by commensal microbiota, while the inner mucus layer is firmly attached to the epithelium and devoid of bacteria. Although the specific bacteria penetrating the inner mucus layer can contact epithelial cells and trigger cancer development, most studies ignore the degree of mucus adhesion at sampling. Therefore, we evaluated whether bacteria adhering to tissues could be identified by removing the outer mucus layer. Our 16S rRNA gene sequencing analysis of 18 surgical specimens of human colorectal cancer revealed that *Sutterella* (*P* = 0.045) and *Enterobacteriaceae* (*P* = 0.045) were significantly enriched in the mucus covering the mucosa relative to the mucosa. *Rikenellaceae* (*P* = 0.026) was significantly enriched in the mucus covering cancer tissues compared with those same cancer tissues. *Ruminococcaceae* (*P* = 0.015), *Enterobacteriaceae* (*P* = 0.030), and *Erysipelotrichaceae* (*P* = 0.028) were significantly enriched in the mucus covering the mucosa compared with the mucus covering cancers. *Fusobacterium* (*P* = 0.038) was significantly enriched in the mucus covering cancers compared with the mucus covering the mucosa. Comparing the microbiomes of mucus and tissues with mucus removed may facilitate identifying bacteria that genuinely invade tissues and affect tumorigenesis.

## Introduction

Colorectal cancer (CRC) is the third most frequent cause of cancer death worldwide^1^ and is influenced by lifestyle^2^ and heredity^3^, among other factors. Recently, increasing evidence has suggested that the gut microbiota are associated with the initiation, progression, and dissemination of CRC^4^. A meta-analysis of eight fecal shotgun metagenomic studies revealed that a core set of 29 species were significantly enriched in CRC metagenomes^5^. Furthermore, it has become clear that the gut microbiota modulate the response to CRC treatment, including chemotherapy, radiotherapy, and immunotherapy^6^.

The colorectal epithelium is covered by mucus, separating bacteria from the colorectal epithelium. Colorectal mucus is produced by goblet cells and organized in two gel layers^7^. Although both layers are composed mainly of a net-like structure of MUC2 mucin, their densities differ substantially^8^. The outer mucus layer is loose, easily removable, and colonized by commensal microbiota. In contrast, the inner mucus layer is much denser, firmly attached to the colon epithelium, and devoid of bacteria^7,8^. This inner mucus layer prevents the commensal microbiota from contacting the colon epithelium and causing chronic inflammation as a result of an immune response^8^.

The association between chronic inflammation and cancer is apparent, and up to 20% of all human cancers are associated with pre-cancerous inflammation^9^. Loss of the barrier function of the inner mucus layer in the colorectum can allow commensal microbiota to have direct contact with colorectal epithelial cells. Under such conditions, some bacteria penetrate the inner mucus layer and adhere to the epithelial cells. Such direct contact of epithelial cells with bacteria can trigger an inflammatory response^10^. Indeed, mice lacking MUC2 mucin develop diarrhea containing blood and ultimately develop colon cancer^11^. Although several bacteria, including *Fusobacterium nucleatum*, are reported to be enriched in human CRC, the results vary from study to study and are sometimes even contradictory^12^.

In most studies assessing the association between microbiota and CRC, mucus and tissue are collected and analyzed altogether. Accordingly, such studies cannot distinguish the bacteria that genuinely adhere to the tissue and may be associated with the initiation and progression of CRC from the other commensal microbiota. Although some studies utilizing an animal model or human colon biopsy samples demonstrate that the microbiota composition of the outer mucus layer differs from that of the inner mucus layer and mucosa^13,14^, studies with human surgical specimens are rare. Gentle suctioning easily removes the outer mucus layer^15^, and the removal of the outer mucus layer can be observed microscopically using Carnoy fixation^16^. Therefore, we evaluated whether bacteria that adhere to tissues can be identified by first removing the outer mucus layer.

## Results

### Comparison of mucus depth

**Figure 1:**
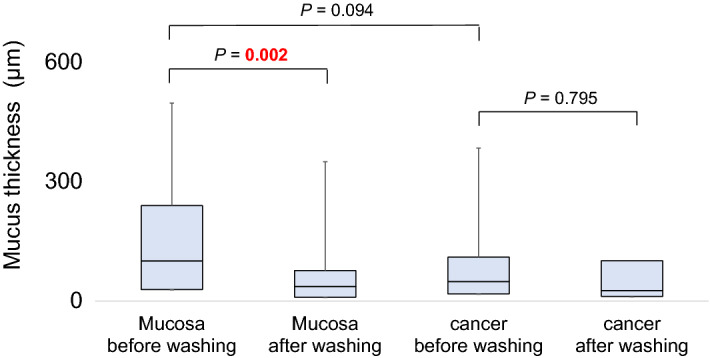
Comparisons of the thickness of the mucus covering the mucosa before and after washing, and of the mucus covering cancer tissue before and after washing by boxplot. Whiskers are extended to the most extreme data point, which is no greater than 1.5 × the interquartile range from the edge of the box in the boxplot. Statistical significance was determined using the Wilcoxon signed-rank test.

First, the depths of the mucus before washing and after washing were compared. A significant difference was found between the median depth of the mucus covering the mucosa before washing and that after washing (100.7 µm vs. 36.8 µm; *P* = 0.002) (Fig. 1). However, no significant difference was found in the median depth of the mucus covering cancer tissue between before washing and after washing (48.9 µm vs. 26.1 µm; *P* = 0.795).

Second, the depths of the mucus covering the mucosa and that covering cancer tissue were compared. Before washing, a marginally significant difference was found between the median depths of mucus covering the mucosa and covering cancer tissue (100.7 µm vs. 48.9 µm; *P* = 0.094); in contrast, after washing, no significant difference was found in this comparison (36.8 µm vs. 26.1 µm; *P* = 0.570).

### Comparison of relative abundance of the microbiome at the phylum level

Samples from feces, mucus covering the mucosa, mucus covering cancer tissue, the mucosa after washing, and cancer tissue after washing were subjected to 16S rRNA gene sequencing to determine the relative abundances of microbiota at the phylum level. The mean relative abundances for each type of sample are shown in Supplementary Fig. S1.

First, the relative abundances in the mucus covering the mucosa and the mucosa after washing were compared (Supplementary Fig. S2). No phyla were significantly enriched in either the mucus or the mucosa. Then, relative abundances in the mucus covering cancer tissue and the cancer tissue itself after washing were compared (Supplementary Fig. S3). No phyla were significantly enriched in either the mucus or the cancer tissue.

Second, relative abundances in the mucus covering the mucosa and cancer tissue were compared (Supplementary Fig. S4). *Proteobacteria* (adjusted *P* = 0.045) was positively enriched in the mucus covering the mucosa. In contrast, *Fusobacteria* (adjusted *P* = 0.040) was significantly enriched in the mucus covering cancer tissue. Then, relative abundances in the mucosa after washing and cancer tissue after washing were compared (Supplementary Fig. S5). *Bacteroides* (adjusted *P* = 0.030) was significantly enriched in the mucosa after washing. No phyla were significantly enriched in cancer tissue after washing.

### Comparison of relative abundance of the microbiome at the genus level

Samples from feces, mucus covering the mucosa, mucus covering cancer tissue, the mucosa after washing, and cancer tissue after washing were subjected to 16S rRNA gene sequencing to determine the relative abundance of microbiota at the genus level. The mean relative abundances for each type of sample are shown in Supplementary Fig. S6.

**Figure 2:**
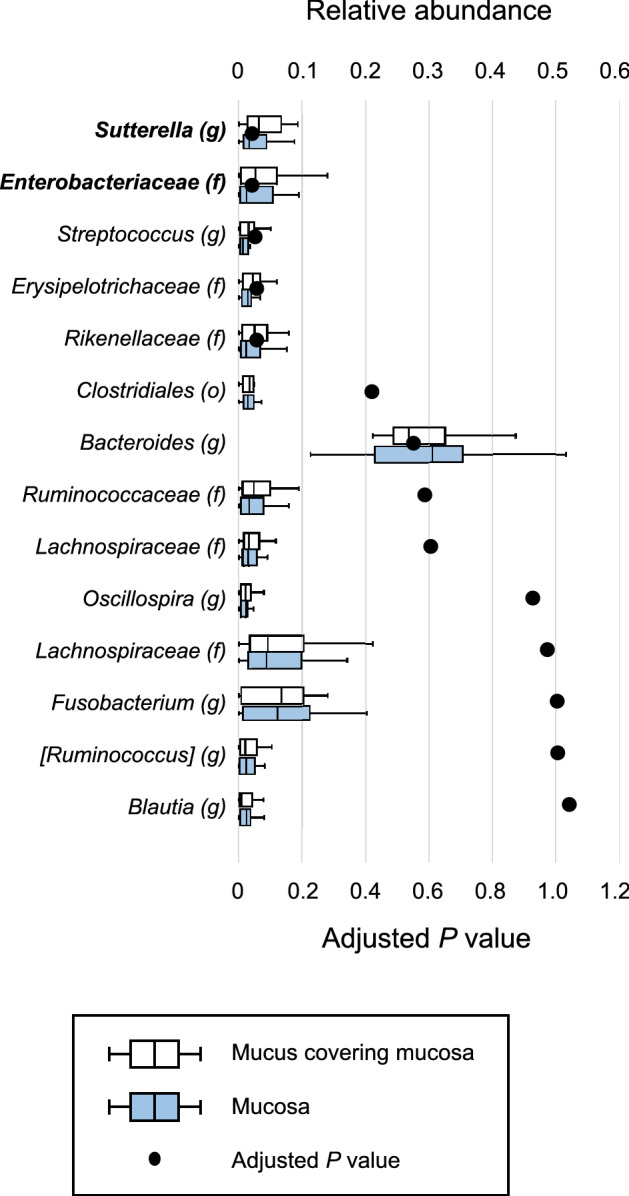
Comparisons of relative abundance in the mucus covering the mucosa and the mucosa after washing at up to the genus level by boxplot. Whiskers are extended to the most extreme data point, which is no greater than 1.5 × the interquartile range from the edge of the box in the boxplot. Statistical significance was determined using the Wilcoxon signed-rank test. The Benjamini–Hochberg method for controlling the false-discovery rate was used for multiple comparisons.

**Figure 3:**
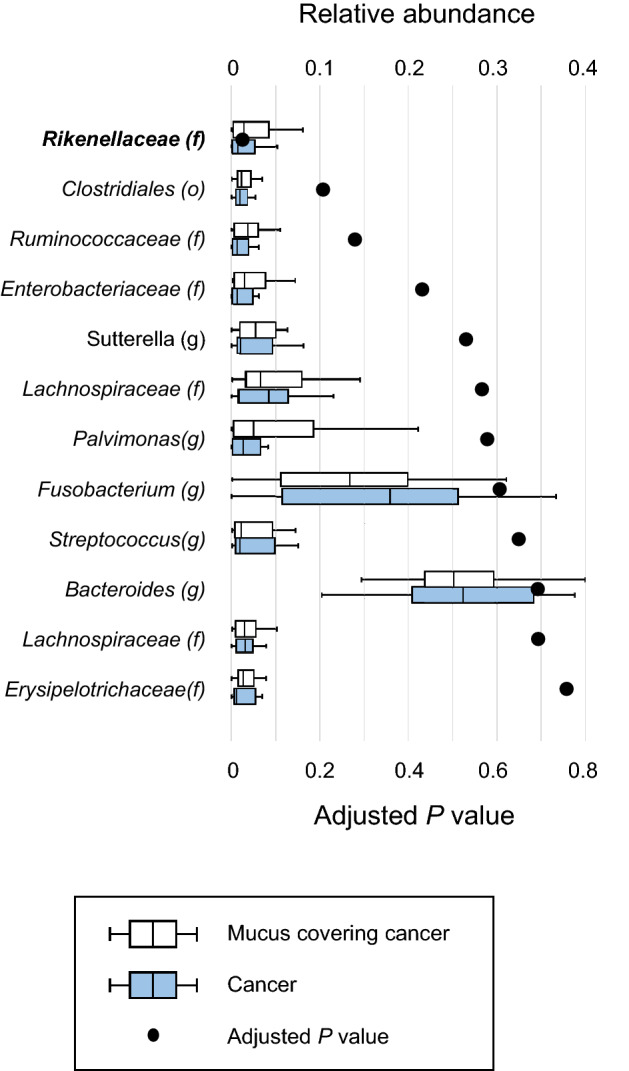
Comparisons of relative abundance in the mucus covering cancer tissue and cancer tissue after washing at up to the genus level by boxplot. Whiskers are extended to the most extreme data point, which is no greater than 1.5 × the interquartile range from the edge of the box in the boxplot. Statistical significance was determined using the Wilcoxon signed-rank test. The Benjamini–Hochberg method for controlling the false-discovery rate was used for multiple comparisons.

First, relative abundances in the mucus covering the mucosa and the mucosa after washing were compared (Fig. 2). The genus *Sutterella* (adjusted *P* = 0.045) and the family *Enterobacteriaceae* (adjusted *P* = 0.045) were significantly enriched in the mucus covering the mucosa. In contrast, no genera or families were significantly enriched in the mucosa after washing. Then, relative abundances in the mucus covering cancer tissue and the cancer tissue itself after washing were compared (Fig. 3). The family *Rikenellaceae* (adjusted *P* = 0.026) was significantly enriched in the mucus covering cancer tissue. In contrast, no genera or families were significantly enriched in the mucosa after washing.

**Figure 4:**
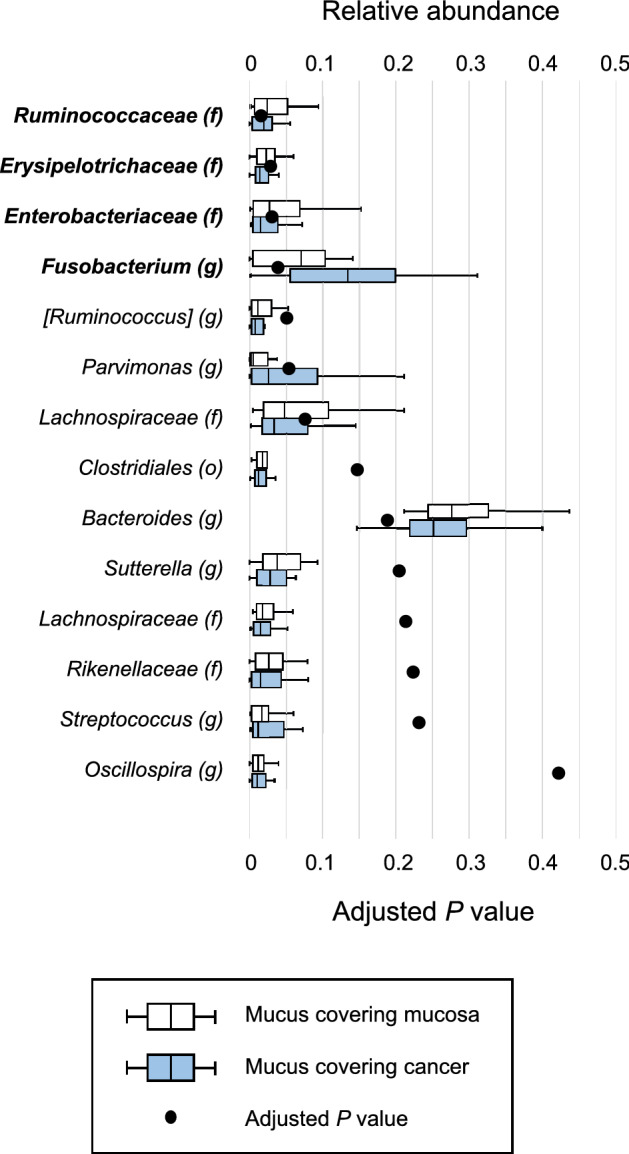
Comparisons of relative abundance in the mucus covering the mucosa and the mucus covering cancer tissue at up to the genus level by boxplot. Whiskers are extended to the most extreme data point, which is no greater than 1.5 × the interquartile range from the edge of the box in the boxplot. Statistical significance was determined using the Wilcoxon signed-rank test. The Benjamini–Hochberg method for controlling the false-discovery rate was used for multiple comparisons.

**Figure 5:**
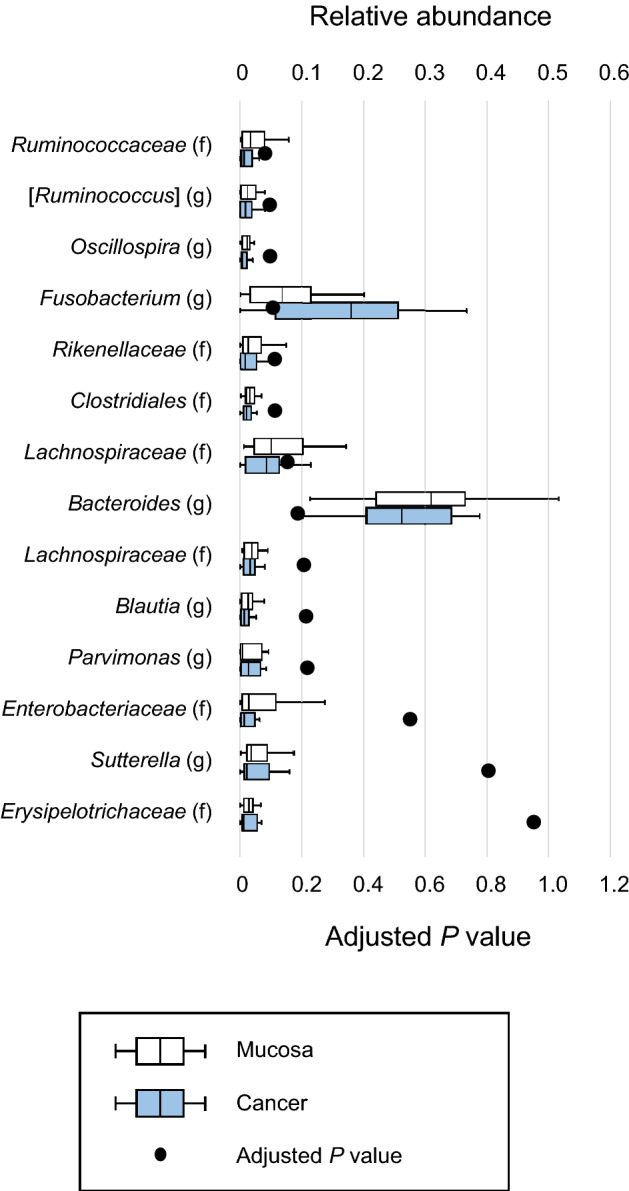
Comparisons of relative abundance in the mucosa after washing and the cancer tissue after washing at up to the genus level by boxplot. Whiskers are extended to the most extreme data point, which is no greater than 1.5 × the interquartile range from the edge of the box in the boxplot. Statistical significance was determined using the Wilcoxon signed-rank test. The Benjamini–Hochberg method for controlling the false-discovery rate was used for multiple comparisons.

Second, relative abundances in the mucus covering the mucosa and that covering cancer tissue were compared (Fig. 4). The families *Ruminococcoaceae* (adjusted *P* = 0.015), *Enterobacteriaceae* (adjusted *P* = 0.030), and *Erysipelotrichaceae* (adjusted *P* = 0.028) were significantly enriched in the mucus covering the mucosa. In contrast, the genus *Fusobacterium* (adjusted *P* = 0.038) was significantly enriched in the mucus covering cancer tissue. Finally, relative abundances in the mucosa after washing and the cancer tissue after washing were compared (Fig. 5). After washing, no genera or families were significantly enriched in either the mucosa or the cancer tissue.

### Microbiota diversity analysis

**Figure 6:**
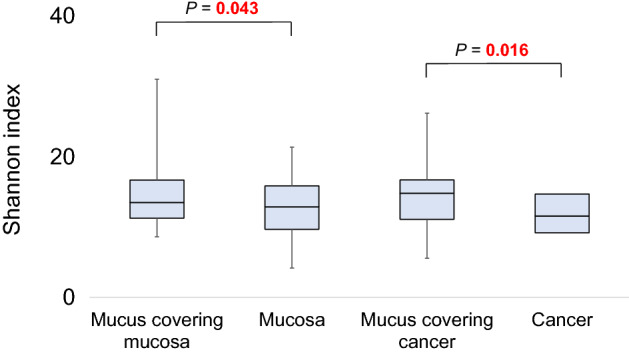
Comparisons of the Shannon index of the mucus covering the mucosa before and after washing, and the mucus covering cancer tissue before and after washing by boxplot. Whiskers are extended to the most extreme data point, which is no greater than 1.5 × the interquartile range from the edge of the box in the boxplot. Statistical significance was determined using the Wilcoxon signed-rank test.

At the genus level, the median Shannon diversity index was higher in the mucus covering the mucosa compared with the mucosa itself after washing (median, 13.5 vs. 12.9; *P* = 0.043) and in the mucus covering cancer tissue compared with the cancer tissue itself after washing (median, 14.8 vs. 11.6; *P* = 0.016) (Fig. 6). In contrast, no significant difference was found between the mucus covering the mucosa and that covering cancer tissue (median, 13.5 vs. 14.8; *P* = 0.711). After washing, a marginally significant difference was found between the mucosa and the cancer tissue (median, 12.9 vs. 11.6; *P* = 0.094). Principal coordinate analyses revealed no remarkable differences between the tissues and the mucus covering those tissues (Supplementary Figs. S7 and S8). No significant differences were found in beta diversity between the mucus covering the mucosa and the mucosa itself after washing (PERMANOVA, *P* = 0.768) and between the mucus covering the cancer tissue and the cancer tissue itself after washing (PERMANOVA, *P* = 0.847).

## Discussion

This study investigated whether bacteria that adhere to tissues in surgical specimens of human CRC can be identified by removing the outer mucus layer, with three notable results. First, the loose mucus layer on tissues could be well removed by high-pressure washing with sterile saline. Second, the relative abundance of some bacteria was significantly decreased in both cancer and mucosal tissues compared with the mucus covering those tissues. Third, the Shannon diversity index was significantly decreased in both cancer and mucosal tissue compared with the mucus covering those tissues.

There are few published evaluations of mucus removal from human colorectal specimens immediately after surgical resection. An in vivo rat model study reported the results of mucus removal by suction with a PE-10 cannula connected to a vacuum suction pump^15^. Another study utilizing human colon biopsy samples reported that the mucus was chemically removed by 500 µl of physiologic saline with 0.016% dithioerythritol and then physically removed by shaking with 500 µl of physiologic saline^17^. In the present study, the mucus was removed with high-pressure washing with sterile saline alone because this method is easy, time-saving, and gentle to tissues. The evaluation with Carnoy fixation in this study revealed that the loose mucus layer covering the mucosa was well removed by this method. Therefore, high-pressure washing using sterile saline is useful for mucus removal of human colorectal specimens after surgical resection.

In this study, the depth of the mucus covering CRC tissue tended to be less than that of the mucus covering the mucosa. Matsuo et al. reported that mucus was rarely identified on the surface of the cancerous or adenoma tissues of most lesions^16^. They also revealed that the inner mucus layer was never identified, even on the surface of goblet cell carcinoma tissue^16^. These results suggest that commensal microbiota can easily have direct contact with CRC or adenoma tissues owing to their lack of a mucus layer. Indeed, Johansson et al. reported that the colonic epithelium of *MUC2*-knockdown (*MUC2* ^−/−^) mice was not covered with mucus, and thus bacteria reached the surface of the epithelium and were also detected deep down into the intestinal crypt^7^. Furthermore, Velcich et al. reported that *MUC2* ^−/−^ mice frequently developed adenomas that progressed to invasive cancer in the small intestine^11^. Thus, the lack of a mucus layer may be associated with the development and progression of CRC through colonizing the commensal microbiome.

Our data suggested that the relative abundance of some bacteria was significantly decreased in cancer or mucosal tissues compared with the mucus covering those tissues. For example, Deng et al. reported that the genus *Sutterella*, which was considerably less abundant in mucosal tissue compared with the mucus covering mucosal tissue in the present study, is associated with CRC patients treated with chemotherapy and is potentially associated with chemoresistance^18^. In contrast, Sun et al. reported that the abundance of the family *Rikenellaceae*, which was significantly less abundant in cancer tissues compared with mucus in the present study, gradually increased during tumorigenesis in a mouse model^19^. Notably, Reunanen et al. suggested that each bacterium adheres to the epithelium through binding to different components of the mucus gel or the epithelial surface^20^. Therefore, analyzing the relative abundance of each bacterium in each sample of mucus and each sample of tissue with mucus removed, as conducted in the present study, may be necessary in order to detect specific bacterial taxa associated with the promotion or suppression of tumorigenesis.

*Fusobacterium* is thought to be a risk factor for the development and progression of CRC^21,22^. *Fusobacterium* adheres to E-cadherin on the surface of epithelial cells with a membrane protein called Fed A and leads to E-cadherin/β-catenin signaling modulation and colorectal carcinogenesis^23^. In our study, the relative abundance of *Fusobacterium* was significantly enriched in the mucus covering cancer compared with the mucus covering the mucosa. However, it was not significantly enriched in the cancer tissue after washing compared with the mucosa after washing. Furthermore, as mentioned above, most CRC tissues are not covered with a mucus layer. These results may imply that *Fusobacterium* preferentially colonizes the surface of tissues, whether each tissue is cancerous or not, rather than the mucus.

Alpha diversity describes within-sample microbial diversity, and several studies have reported that a decrease in microbial alpha diversity is positively associated with CRC. Ahn et al. reported that CRC patients had decreased alpha diversity, including Shannon’s diversity, compared with controls, based on fecal 16S rRNA gene sequencing data^24^. Yoon et al. also reported that the alpha diversity of CRC tissue is lower than that of normal mucosa and adenoma tissues, based on 16rRNA gene sequencing of biopsy samples^25^. However, Araújo-Pérez et al. reported that bacterial alpha diversity was significantly higher in rectal swab samples than in rectal biopsies from healthy adults^26^. In this study, the Shannon diversity index was significantly decreased in both cancer and mucosa samples compared with the mucus covering those tissues. Although our findings are compatible with those previous reports, further research is needed to determine whether the differences in diversity are cancer related or sampling related.

The limitations of our study include the small number of samples and the lack of a cohort randomized on the basis of factors such as tumor location, histologic type, stage of cancer progression, and interindividual differences in the gut microbiome. Preoperative mechanical bowel preparation or prophylactic antibiotic administration might also affect microbiome composition and diversity. There was no non-CRC control group in our study because it is difficult to obtain healthy colon specimens. Furthermore, the microbiota analysis in our study relied on a comparison of relative rather than absolute taxon abundance, which may mask real differences in the total number of bacteria in different sample types. However, separate sampling of mucus and tissues with mucus removed, especially from fresh surgical specimens, is rare and suggests that sampling methods may significantly affect the results of microbiome analysis.

By comparing the microbiomes of mucus and tissues with mucus removed, we demonstrate the possibility of identifying bacterial taxa that genuinely invade colorectal tissues and promote or suppress tumorigenesis.

## Methods

### Patients

This prospective analysis was performed in accordance with the Helsinki Declaration. The Ethics Committee of the School of Medicine, Fujita Health University, approved the study protocol (HM19-205), and written informed consent was obtained from all patients. A total of 18 CRC patients who underwent primary tumor resection between February 2020 and January 2021 at the Fujita University Hospital were enrolled. Patients who received preoperative chemotherapy or radiotherapy were excluded. Patients with familial adenomatous polyposis or inflammatory bowel disease were excluded. None of the patients had received antibiotics within a month before surgery. The clinicopathological characteristics of the 18 patients are shown in Supplementary Table S1.

### Sampling method

The day before surgery, the bowel was cleaned with 34 g of magnesium citrate. Fecal samples were collected immediately after starting the oral administration of magnesium citrate at the first defecation. The fecal samples were stored in guanidine thiocyanate solution (100 mM Tris–HCl [pH 9.0], 40 mM EDTA, and 4 M guanidine thiocyanate) at room temperature^27^. All patients were administered cefmetazole just before the start of surgery. After curative resection of CRC, the intestinal wall of the specimen was cut longitudinally with sterile scissors and gloves, and the lumen was exposed within 15 min after resection. Then, the loose outer mucus layer covering the cancer and non-cancerous mucosa was collected by rubbing the specimens with a sterile cotton swab. The collection point of non-cancerous mucosa was proximally 5 cm away from the tumor. For tumors located in the cecum, the collecting point of non-cancerous mucosa was 5 cm distal from the cancer. Then, the loose outer mucus layer covering the cancer and mucosa at the planned collection site was removed by high-pressure washing with 500 ml of sterile saline using an 18-gauge needle and a 50-ml syringe. After the washing solution on the tissues was gently absorbed with a sterile gauze to prevent contamination, the washed cancer tissue and non-cancerous mucosa were collected with sterile scissors and forceps. All layers of the cancer tissue and non-cancerous lesion before and after washing were also collected for Carnoy fixation at similar points. The samples were stored after being snap-frozen in a 3.0-ml Tissue Tube (FCR&BIO, Kobe, Japan) at − 80℃.

### Carnoy fixation, Alcian blue–periodic acid Schiff staining, and measurement of mucus depth

**Figure 7:**
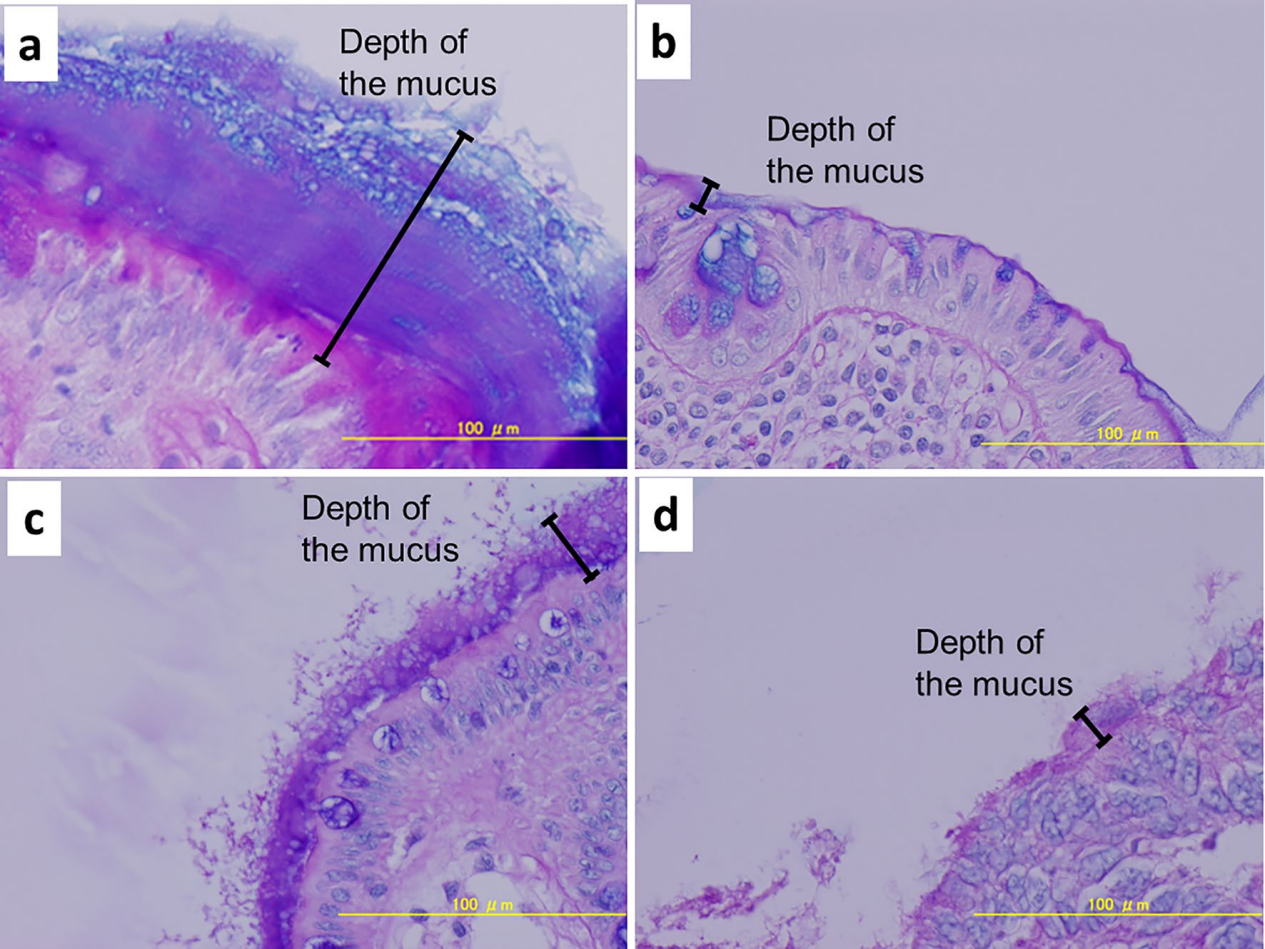
Measurement of the depth of mucus under microscopy using samples prepared by Carnoy fixation and staining with Alcian blue–periodic acid Schiff, (**a**) Non-cancerous lesion before washing, (**b**) Non-cancerous lesion after washing, (**c**) Cancer tissue before washing, and (**d**) Cancer tissue after washing.

Each specimen comprising both cancer tissue and non-cancerous lesion was fixed in nonaqueous Carnoy solution^16^ for 2 h and then processed and embedded into paraffin blocks by standard techniques. Five-micron-thick sections were prepared and stained with Alcian Blue–periodic acid Schiff. The entire section including the mucus layer was first viewed at low-power magnification to identify the area containing the maximum depth of mucus. Then, the mucus depth for each section was measured twice on different days under a microscope using a × 20–40 objective lens (Fig. 7), and the average depth was used for analysis. One surgical pathologist (YT) blinded to all clinical details assessed each section.

### 16S rRNA gene sequencing

We performed 16S rRNA gene sequencing as described in a previous report^28^. The collected samples were sent to the laboratory facilities of Cykinso Inc. (Tokyo, Japan). DNA was extracted from feces, mucus, and tissue samples using an automated DNA extraction machine (GENE PREP STAR PI-480; Kurabo Industries Ltd., Osaka, Japan) according to the manufacturer’s instructions. For each sample, 16S rRNA gene sequencing was performed using Mykinso technology developed by Cykinso Inc., which includes DNA extraction and subsequent 16S rRNA paired-end sequencing using the Illumina MiSeq platform^29^. The FASTQ file thus obtained was processed to join the forward and reverse reads into a single read per sample by using fastq-join^30^ with the default settings. Next, low-quality sequences were excluded using QIIME version 1.9^31^, and chimeric sequences were removed using USEARCH^32^. Relative abundance was calculated after detecting OTUs at 97% identity for the filtered sequence data by using QIIME’s pick_open_reference_otus command.

### Contaminant filtering and re-calculation of relative abundance

We performed contaminant filtering and re-calculation of relative abundance as described in a previous report^28,33^. Sequence data corresponding to species that matched known contaminant species were filtered out, and then we re-calculated the relative abundance of the remaining species to be normalized to 1.0. The relative abundance at the upstream taxonomy levels was re-estimated based on the re-calculated relative abundance at the species level.

### Statistical analysis

The mucus depth was compared between before and after washing by Wilcoxon signed-rank test to evaluate whether mucus was removed by high-pressure washing with sterile saline. Comparisons of the relative abundances of taxa between mucus and washed tissue samples were also assessed by the Wilcoxon signed-rank test. Only phyla or genera with a median relative abundance > 0.01 in at least one group were selected for this analysis. The Benjamini–Hochberg method for controlling the false-discovery rate was used to account for multiple comparisons. The Shannon diversity index was used to evaluate alpha diversity and was compared between mucus and washed tissue samples once again by the Wilcoxon signed-rank test. PERMANOVA comparisons of beta diversity were performed using the R package vegan^34^. Statistical analyses were performed using IBM SPSS Statistics 27 (IBM Japan Inc., Tokyo, Japan). *P* values less than 0.05 were considered statistically significant.

## Supplementary Information

Supplementary Information.

## Data Availability

Data and material will be made available upon reasonable request and with the approval of the corresponding author. The datasets generated and/or analyzed in the present study are available in the DDBJ DRA repository PRJDB13561^35^　(https://ddbj.nig.ac.jp/resource/bioproject/PRJDB13561). The patient-to-sample number matching table for 16S rRNA gene sequencing is shown in Supplementary Table S2.
