# Supplementary material for: Differential analysis of microbiomes in mucus and tissues obtained from colorectal cancer patients
Source: Sci Rep. 2022 Oct 28;12:18193. doi: 10.1038/s41598-022-21928-4 (PMC9616824; doi:10.1038/s41598-022-21928-4)
Supplement: Supplementary file 1 — Supplementary Information. [file 41598_2022_21928_MOESM1_ESM.pdf]

Supplementary information for

Differential analysis of microbiomes in mucus and tissues obtained from colorectal cancer patients

Yosuke Tajima, MD, PhD, Shujiro Okuda, PhD, Tsunekazu Hanai, MD, PhD, Junichiro Hiro, MD, PhD, Koji Masumori, MD, PhD, Yoshikazu Koide, MD, PhD, Tadahiro Kamiya, MD, PhD, Yeongcheol Cheong, MD, PhD, Gaku Inaguma, MD, Yoshifumi Shimada, MD, PhD, Toshifumi Wakai, MD, PhD, Hayato Takihara, PhD, Shingo Akimoto, PhD, Hiroshi Matsuoka, MD, PhD, Ichiro Uyama, MD, PhD, Koichi Suda, MD, PhD

Contents

Supplementary Figure S1. Relative abundance of bacteria in feces, mucus, and tissues after washing at the phylum level.

Supplementary Figure S2. Comparisons of relative abundance in the mucus covering the mucosa and the mucosa itself after washing at the phylum level by boxplot. Whiskers are extended to the most extreme data point, which is no greater than 1.5× the interquartile range from the edge of the box in the boxplot. Statistical significance was determined using the Wilcoxon signed-rank test. The Benjamini–Hochberg method for controlling the false-discovery rate was used for multiple comparisons.

Supplementary Figure S3. Comparisons of relative abundance in the mucus covering cancer tissue and the cancer tissue itself after washing at the phylum level by boxplot. Whiskers are extended to the most extreme data point, which is no more than 1.5× the interquartile range from the edge of the box in the boxplot. Statistical significance was determined using the Wilcoxon signed-rank test. The Benjamini–Hochberg method for controlling the false-discovery rate was used for multiple comparisons.

Supplementary Figure S4. Comparisons of relative abundance in the mucus covering the mucosa and the mucus covering cancer tissue at the phylum level by boxplot. Whiskers are extended to the most extreme data point, which is no more than 1.5× the interquartile range from the edge of the box in boxplot. Statistical significance was determined using the Wilcoxon signed-rank test. The Benjamini–Hochberg method for controlling the false-discovery rate was used for multiple comparisons.

Supplementary Figure S5. Comparisons of relative abundance in the mucosa after washing and cancer tissue after washing at the phylum level by boxplot. Whiskers are extended to the most extreme data point, which is no greater than 1.5× the interquartile range from the edge of the box in the boxplot. Statistical significance was determined using the Wilcoxon signed-rank test. The Benjamini–Hochberg method for controlling the false-discovery rate was used for multiple comparisons.

Supplementary Figure S6. Relative abundance of bacteria in feces, mucus, and tissues after washing at the genus level.

Supplementary Figure S7. Principal coordinate analysis comparing the abundance of the mucus covering the mucosa and the mucosa itself.

Supplementary Figure S8. Principal coordinate analysis comparing the abundance of the mucus covering cancer tissue and the cancer tissue itself.

Supplementary Figure S1

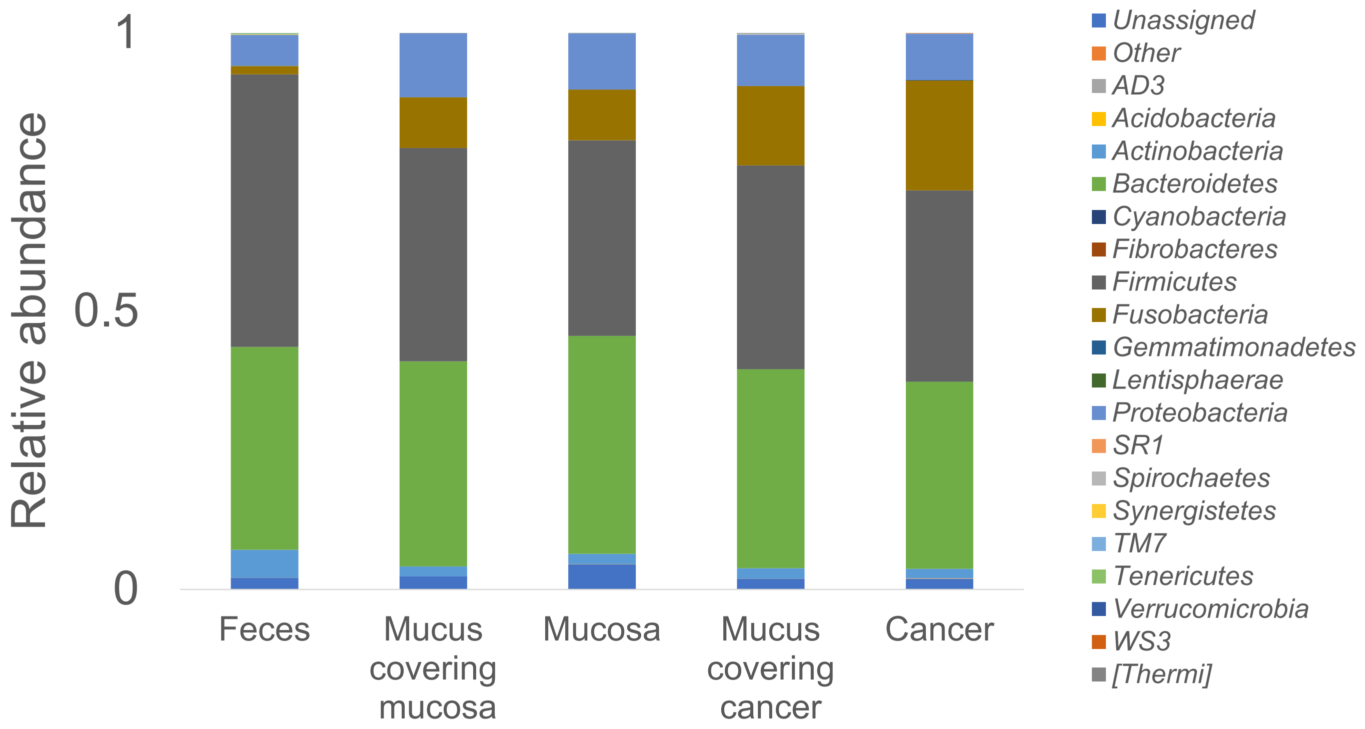

Supplementary Figure S2

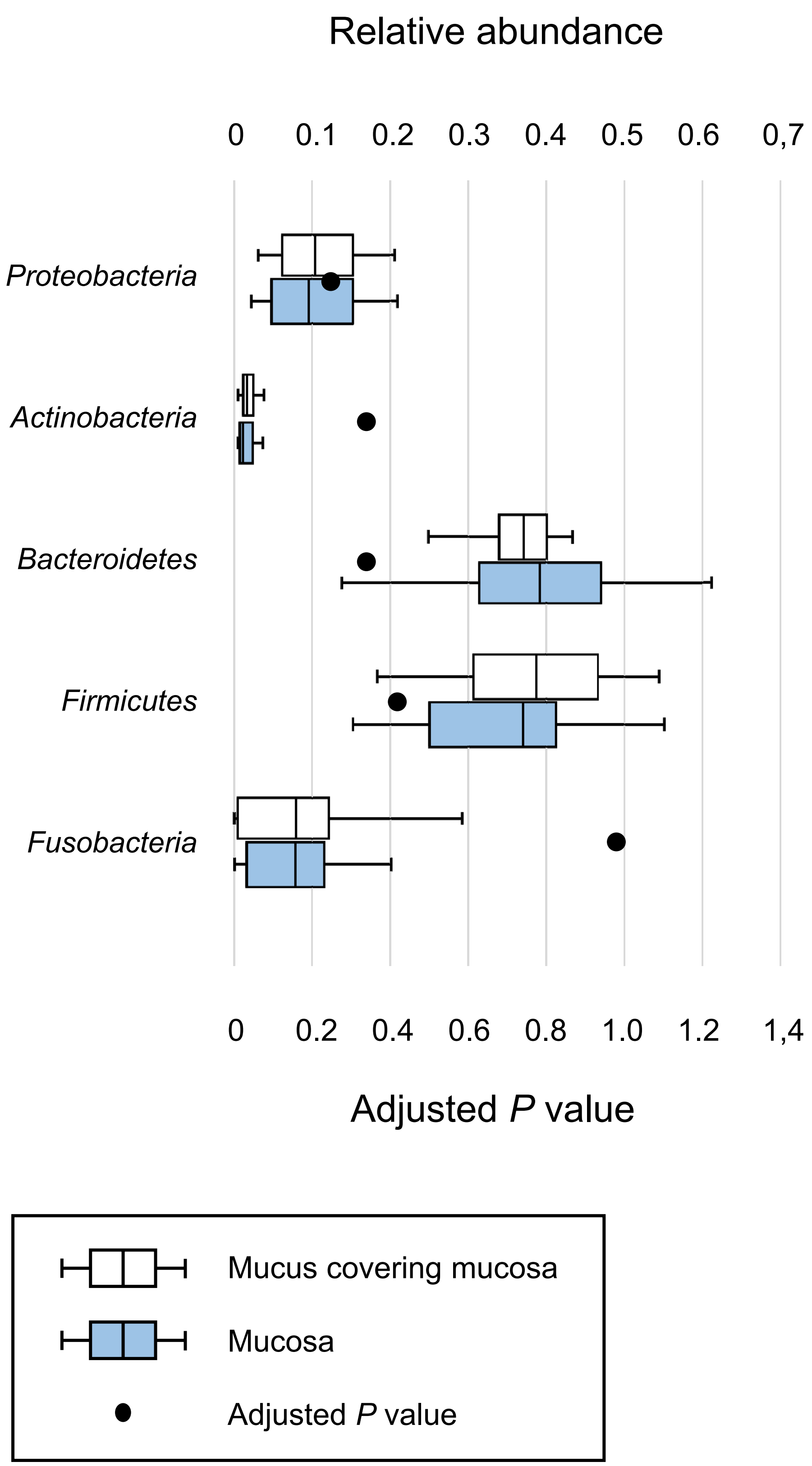

Supplementary Figure S3

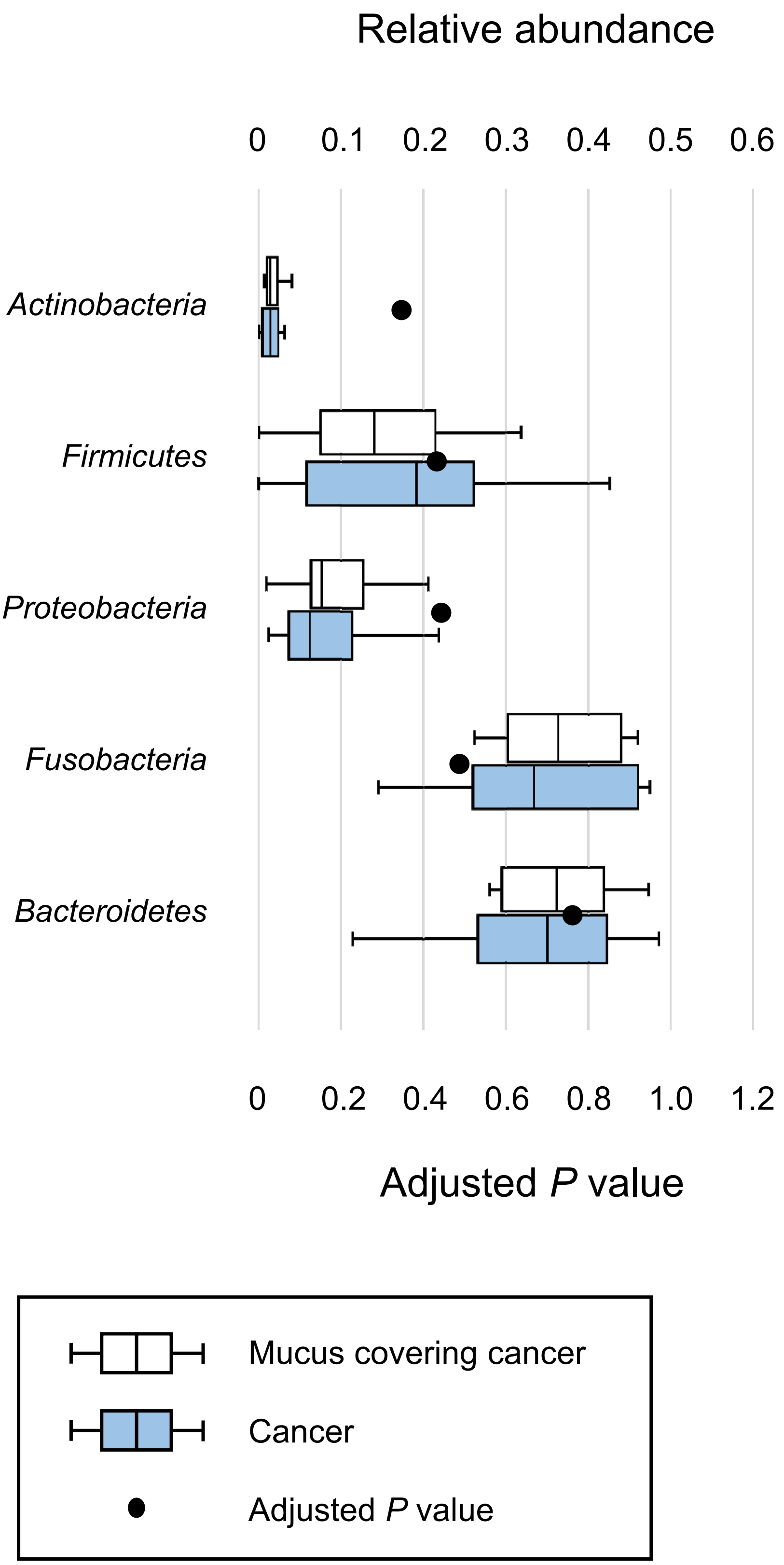

Supplementary Figure S4

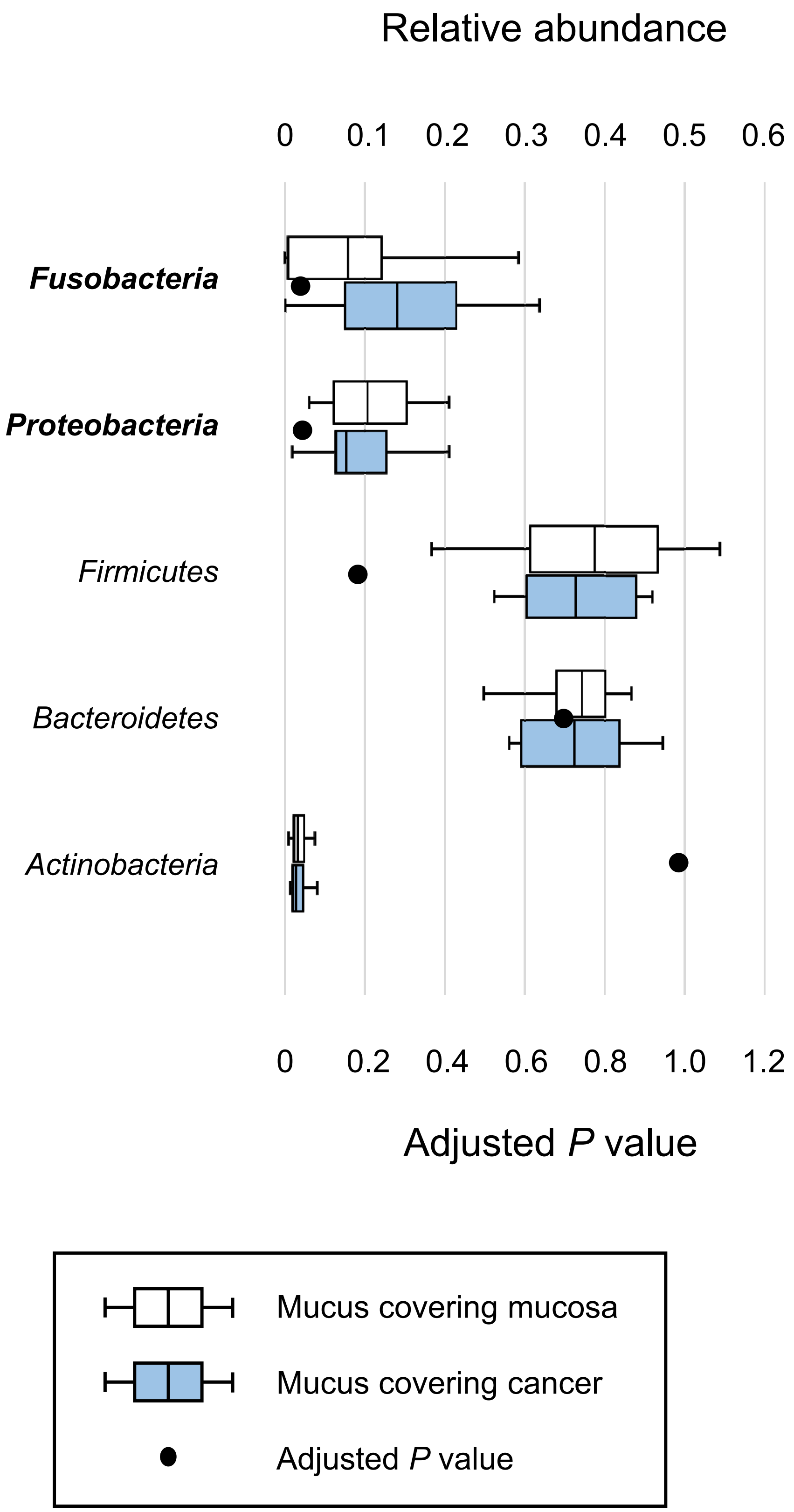

Supplementary Figure S5

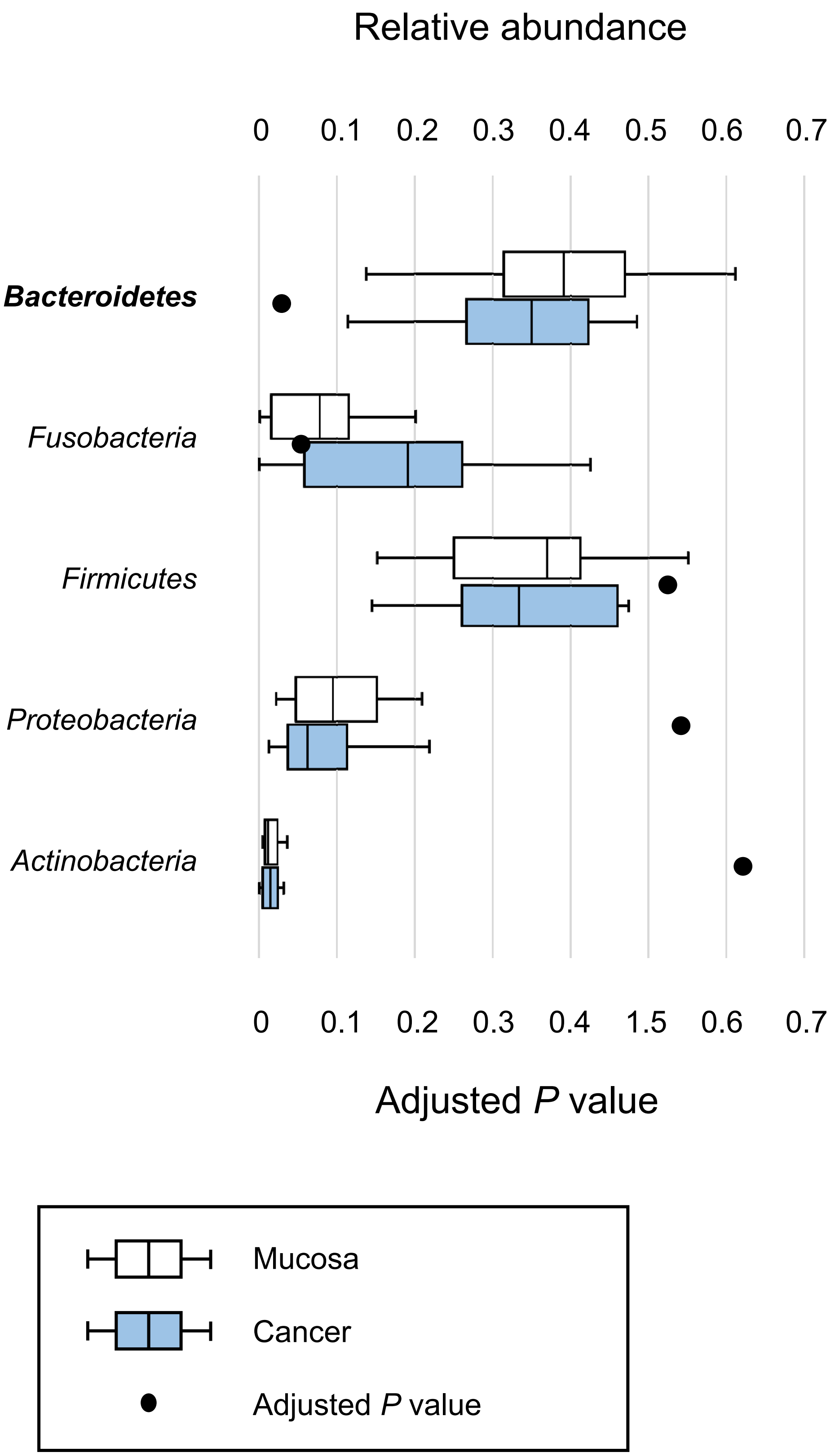

Supplementary Figure S6

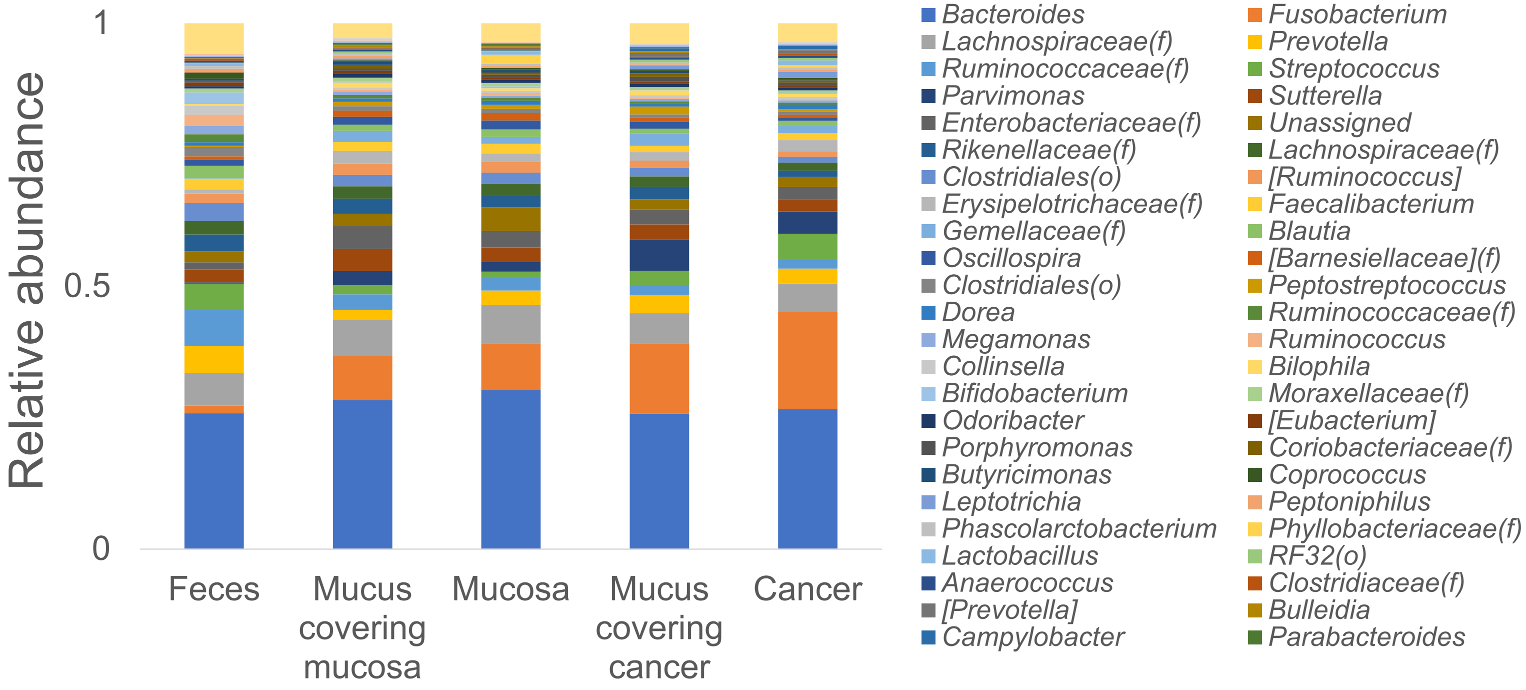

Supplementary Figure S7

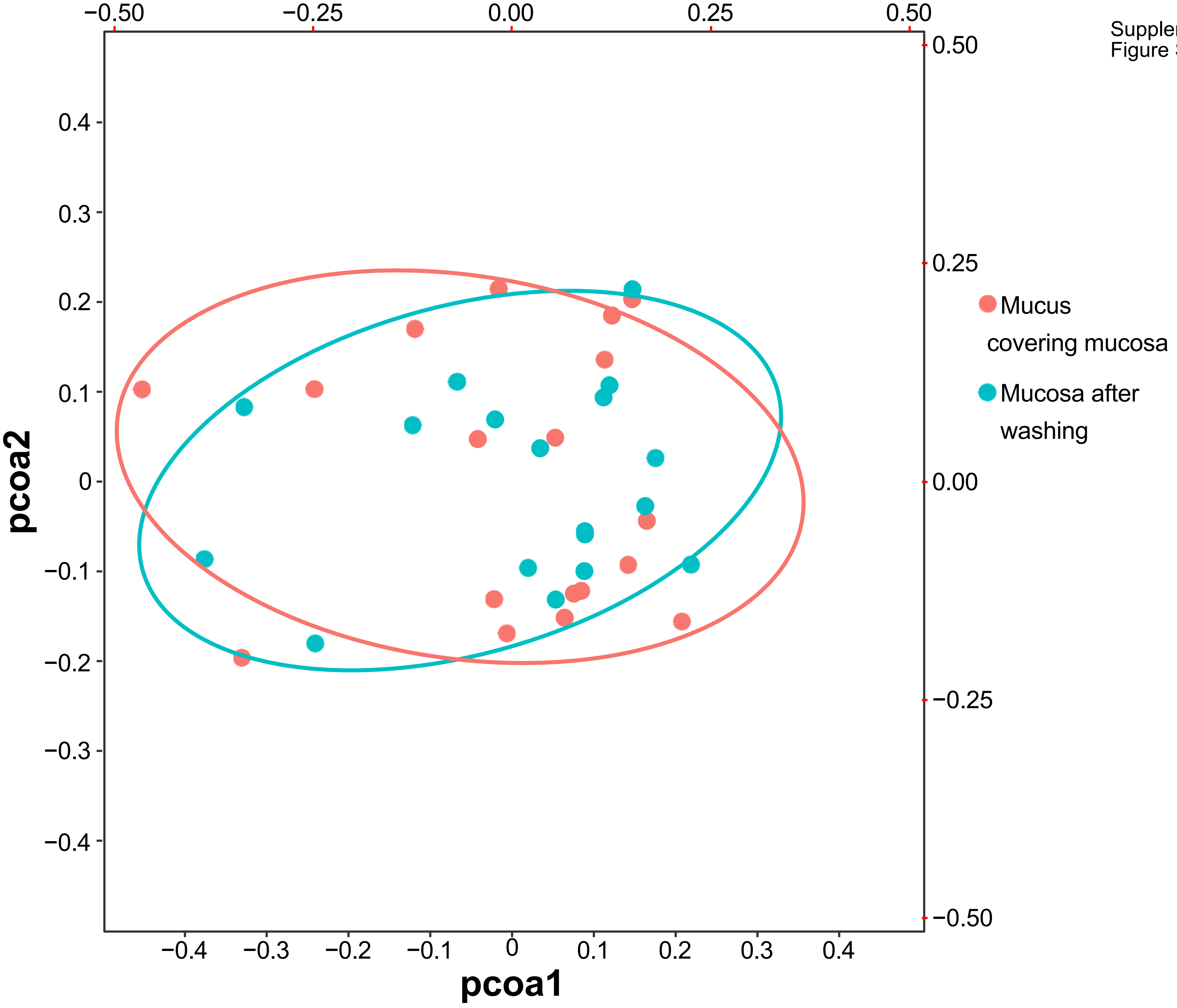

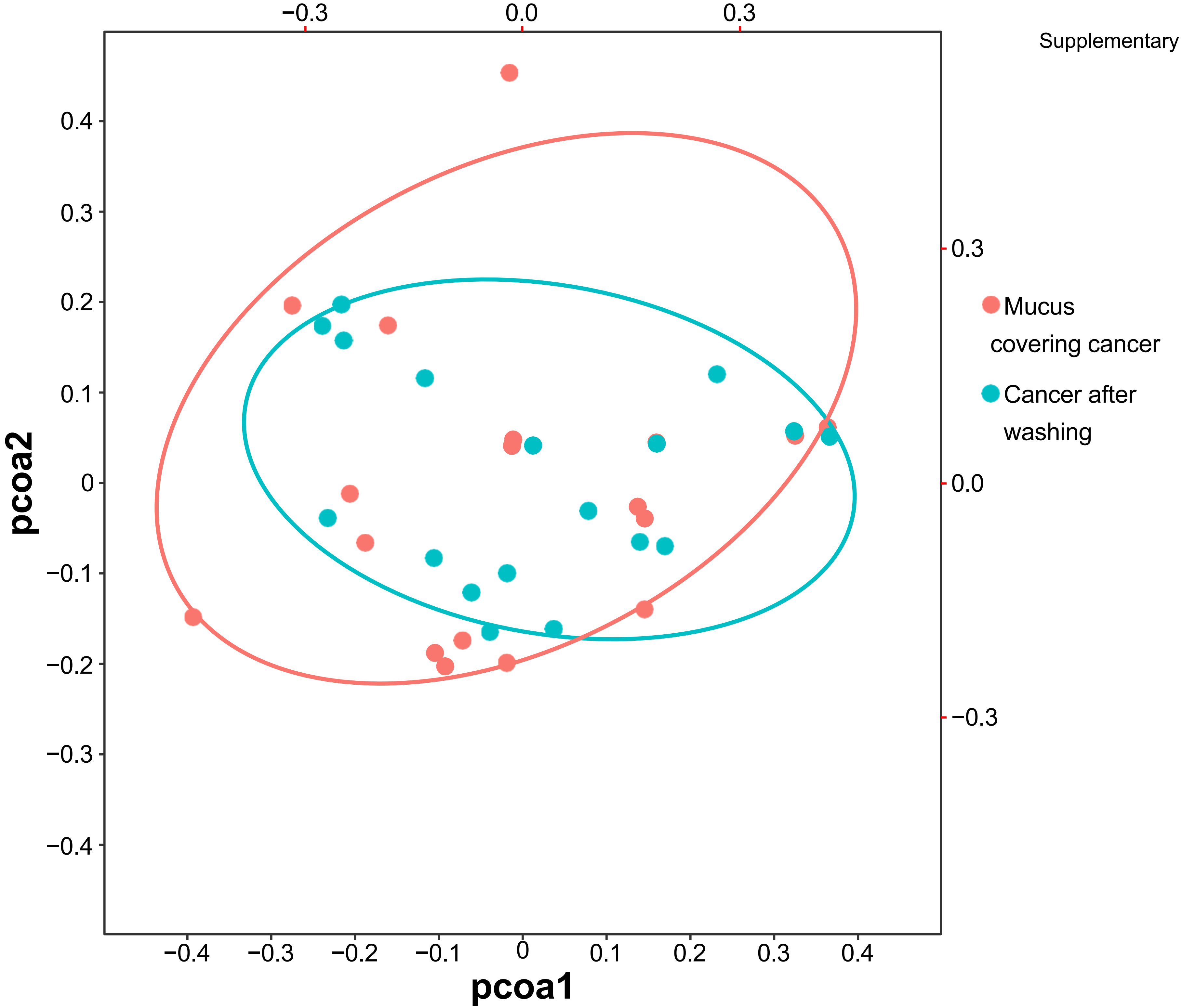
−0.3
0.0
0.3
0.4
0.3
0.2
0.1
0
−0.1
−0.2
−0.3
−0.4
pcoa2
0.3
0.0
−0.3
Mucus covering cancer
Cancer after washing
−0.4
−0.3
−0.2
−0.1
0
0.1
0.2
0.3
0.4
pcoa1

Supplementary Table S1. Clinicopathological characteristics of 18 patients.

Gender	
Male	12
Female	6
Age (years)	
<65	5
≥65	13
Tumor location	
Right-side colon	9
Left-side colon	5
Rectum	4
Tumor size (mm)	
<50	6
≥50	12
Histopathological grading	
G1, G2	16
G3	2
Tumor stage	
T1, T2	3
T3, T4	15
Lymphatic invasion	
Absence	1
Presence	17
Venous invasion	
Absence	1
Presence	17
Nodal involvement	
N0	7
N1, N2	11
Distant metastasis	
M0	14
M1	4

Supplementary Table S2. Patient-to-sample number matching table for 16S rRNA gene sequencing.

Patient number	Sample number				
	feces	Mucus covering mucosa	Mucus covering cancer	Mucosa	Cancer
FCS001	018001602591.R1.R2	200514.tajima.001NTbefore.R1.R2	200514.tajima.001Tafter.R1.R2	200514.tajima.001NTbefore.R1.R2	200514.tajima.001Tbefore.R1.R2
FCS002	017001502590.R1.R2	200514.tajima.002NTbeforeT.R1.R2	200514.tajima.002TafterT.R1.R2	200514.tajima.002NTbeforeT.R1.R2	200514.tajima.002TbeforeT.R1.R2
FCS003	018000302599.R1.R2	200514.tajima.003NTbefore.R1.R2	200514.tajima.003Tafter.R1.R2	200514.tajima.003NTbefore.R1.R2	200514.tajima.003Tbefore.R1.R2
FCS004	015000202597.R1.R2	200717.tajima.004NTbefore.R1.R2	200717.tajima.004Tafter.R1.R2	200717.tajima.004NTbefore.R1.R2	200717.tajima.004Tbefore.R1.R2
FCS005	014000502593.R1.R2	200717.tajima.005NTbefore.R1.R2	200717.tajima.005Tafter.R1.R2	200717.tajima.005NTbefore.R1.R2	200717.tajima.005Tbefore.R1.R2
FCS006	013000802599.R1.R2	200717.tajima.006NTbefore.R1.R2	200717.tajima.006Tafter.R1.R2	200717.tajima.006NTbefore.R1.R2	200717.tajima.006Tbefore.R1.R2
FCS007	010000702597.R1.R2	200717.tajima.007NTbefore.R1.R2	200717.tajima.007Tafter.R1.R2	200717.tajima.007NTbefore.R1.R2	200717.tajima.007Tbefore.R1.R2
FCS008	018009502563.R1.R2	200717.tajima.008NTbefore.R1.R2	200717.tajima.008Tafter.R1.R2	200717.tajima.008NTbefore.R1.R2	200717.tajima.008Tbefore.R1.R2
FCS009	013001102596.R1.R2	200717.tajima.009NTbefore.R1.R2	200717.tajima.009Tafter.R1.R2	200717.tajima.009NTbefore.R1.R2	200717.tajima.009Tbefore.R1.R2
FCS011	017000602595.R1.R2	200717.tajima.011NTbefore.R1.R2	200717.tajima.011Tafter.R1.R2	200717.tajima.011NTbefore.R1.R2	200717.tajima.011Tbefore.R1.R2
FCS012	019010002562.R1.R2	200717.tajima.012NTbefore.R1.R2	200717.tajima.012Tafter.R1.R2	200717.tajima.012NTbefore.R1.R2	200717.tajima.012Tbefore.R1.R2
FCS013	012009902567.R1.R2	210108.tajima.013NTbefore.R1.R2	210108.tajima.013Tafter.R1.R2	210108.tajima.013NTbefore.R1.R2	210108.tajima.013Tbefore.R1.R2
FCS014	010009702565.R1.R2	210108.tajima.014NTbeforeA.R1.R2	210108.tajima.014TafterA.R1.R2	210108.tajima.014NTbeforeA.R1.R2	210108.tajima.014TbeforeA.R1.R2
FCS015	011009802566.R1.R2	210108.tajima.015NTbefore.R1.R2	210108.tajima.015Tafter.R1.R2	210108.tajima.015NTbefore.R1.R2	210108.tajima.015Tbefore.R1.R2
FCS016	016009302561.R1.R2	210108.tajima.016NTbefore.R1.R2	210108.tajima.016Tafter.R1.R2	210108.tajima.016NTbefore.R1.R2	210108.tajima.016Tbefore.R1.R2
FCS018	012001002595.R1.R2	210108.tajima.018NTbefore.R1.R2	210108.tajima.018Tafter.R1.R2	210108.tajima.018NTbefore.R1.R2	210108.tajima.018Tbefore.R1.R2
FCS019	017009402562.R1.R2	210108.tajima.019NTbefore.R1.R2	210108.tajima.019Tafter.R1.R2	210108.tajima.019NTbefore.R1.R2	210108.tajima.019Tbefore.R1.R2
FCS020	011003303582.R1.R2	210108.tajima.020NTbefore.R1.R2	210108.tajima.020Tafter.R1.R2	210108.tajima.020NTbefore.R1.R2	210108.tajima.020Tbefore.R1.R2
